# Efficacy of differential reinforcement of other behaviors therapy for tic disorder: a meta-analysis

**DOI:** 10.1186/s12883-023-03501-2

**Published:** 2024-01-02

**Authors:** Zakaria Ahmed Mohamed, Yang Xue, Miaoshui Bai, Hanyu Dong, Feiyong Jia

**Affiliations:** Department of Developmental and Behavioral Pediatrics, The First Hospital of Jilin University, Changchun, China

**Keywords:** Efficacy, DRO, Tic disorder, Therapy

## Abstract

**Introduction:**

Recently, studies on behavioral tic suppression techniques have gained popularity as opposed to pharmacological alternatives that often have potentially dangerous side effects. Differential Reinforcement of Other Behaviors therapy (DRO) is one such behavioral technique whose efficacy in tic suppression has been experimentally demonstrated albeit in studies with very few patients, and lacking statistical power. Here, we conducted a meta-analysis of these studies to improve their overall power and explore whether DRO intervention is really effective for tic suppression.

**Materials and methods:**

PubMed, Embase, PsycINFO, and Cochrane Library were searched from inception to August 30, 2023. Only original interventional studies that examined the efficacy of DRO for tic suppression were included.

**Results:**

A total of 8 no control interventional studies involving 79 children with tic disorders were recruited. Most of the children had moderate tic severity. The pooled mean Yale Global Tic Severity Scale (YGTSS) score was 24.64 (95% CI: 21.99 – 30.12, *p* =  < 0.00001, *I*^*2*^ = 87%). In terms of efficacy of the DRO technique for tic suppression, the results showed that DRO was effective in reducing tic frequency among the children. The pooled standardized mean difference (SMD) was -10.25 (95% CI: -14.71 – -5.79, *p* =  < 0.00001) with *I*^*2*^ = 94%.

**Conclusion:**

In conclusion, this study revealed that DRO is potentially an effective tic suppression technique for temporarily managing tic disorder. It also showed that DRO could be employed for both moderate and severe tic disorders. However, the technique bears crucial limitations that limit its implementation outside of experimental settings. More studies are needed to address these limitations and improve its applicability in the real world.

**Supplementary Information:**

The online version contains supplementary material available at 10.1186/s12883-023-03501-2.

## Introduction

Tic disorders (TD) are neuropsychiatric disorders of childhood-onset characterized by sudden, rapid, repetitive, nonrhythmic movements or vocalizations called “tics” [1]. Evidence from recent anatomical, functional, and lesion experiments indicate that Tic disorder is a network problem resulting from disturbed interplay within and between large scale brain networks as opposed to localized dysfunction of specific single brain regions [2]. It is a result of the failure of the cortico-striato-thalamo-cortical circuits to prevent somatosensory urges and associated motor activities that constitute tic behaviors [3–6]. A network of frontal areas, along with the basal ganglia, insula and cerebellum, have been strongly linked to tic behavior, suggesting a crucial role for the basal ganglia-cerebellar-thalamo-cortical system in the pathophysiology of tics [7–9]. Importantly, tics are considered a focal excitatory abnormality in the striatum, causing increased inhibition of the globus pallidus internus, leading to a disinhibition of the thalamic and—in turn -cortical neurons [9, 10]. A role for the cerebellum has also been suggested, hypothesized as a disynaptic link from the basal ganglia to the cerebellum [11] although its details need further studies.

Occurrence of Tics is often preceded by a “premonitory urge,” defined as a feeling of discomfort (e.g., a sensation like an itch or pressure, or a sense that one must tic), which is then temporarily relieved when the tic occurs [12]. This reflects a deficient inhibitory control over the motor response to premonitory urge, which evidence suggest is due to disruptions in movement-regulation functions mediated by the basal ganglia [13]. Further evidence suggest that increased activity in the primary somatosensory cortex, putamen, and amygdala/hippocampus of the cortico-striato-thalamo-cortical circuit in patients with tic disorder relative to normal individuals represent activity associated with “premonitory urge” that act a trigger for tic behaviors [14]. This disruption has been shown under both laboratory and natural conditions to be modulated by environmental and behavioral factors such as being in public, stress, anxiety, and anger among others [15–18].

Based on the consensus that behavioral factors affect tic maintenance, a number of behavioral interventions for tic suppression have been developed, and experimentally shown to be effective [19, 20]. Differential reinforcement of other behaviors (DRO) is one of such interventions termed contingency management [15, 21–23]. It involves actively and positively reinforcing tic suppression in a subject by providing small reinforcers (e.g., tokens or small amounts of money) in exchange for progressively longer periods of successful tic suppression [24]. This reinforces a competing response that is performed prior to the occurrence of the tic, thus interrupting it [25]. According to Milternberger and colleagues, [26] this process involves contraction of muscles that are antagonistic to the premonitory urges that brings about tics, and so reinforcing the competitive response strengthens it until its performance becomes habitual. Woods and colleagues, [27] suggested that competing responses (e.g. those in DRO) may not necessarily be antagonistic to the urge but rather provide strategies for tic suppression that allows the premonitory urge to habituate, hence reducing the occurrence of tics. DRO’s application in tic suppression is supported by a number of case studies and single group experiments [15, 17, 28]. However, it has yet to be evaluated in large randomized controlled trails.

DRO is an easy to implement tic suppression strategy. It can be implemented by both experienced and inexperienced behavioral scientists. Moreover, its reward system can be modelled on real world threats to a child with tics e.g. the promise of avoidance of teasing from peers, or stares by strangers or promise to engage in sports that could have been prevented by tics [16]. Despite these obvious advantages, the technique has not been widely explored for the management of tics. There is convincing evidence from single group studies and case reports of the efficacy of DRO for managing tic disorders; however, most if not all of the studies lacked controls and involved just a handful of subjects, which significantly limits the strength of their findings. Lack of large scale randomized controlled trials further limits the strength of the available evidence. As a result, the real effect of DRO intervention on tic suppression remains unclear. In this study, we sought to synthesize evidence in support of DRO intervention for tic disorders by combining all the before and after intervention studies that assessed DRO intervention in tics. We utilized the number of tics per minute before and after intervention as a severity score to determine efficacy of DRO in tic suppression. We hoped this meta-analysis would provide a stronger case for future large scale randomized controlled trials on efficacy of DRO for tic disorders.

## Methods

### Search strategy and selection criteria

This study was conducted according to the 2020 Preferred Reporting Items for Systematic reviews and Meta-Analyses (PRISMA) updated guideline for reporting systematic reviews and meta-analyses [29]. PubMed, Embase, PsycINFO, and Cochrane Library were searched from inception to August 30, 2023.The literature search was conducted using Medical Subject Headings (MeSH terms) and free-text words that might appear in the titles and/or abstracts of the relevant papers: "TD", "tic disorder", "Tourette syndrome", "tics", "tic Suppression", "tic control" "tic reduc*", "Behavioral therapy", "DRO", "Differential Reinforcement of Other Behaviors therapy”, "child*". Detailed search terms and strategy is presented in Supplementary material 1. Additionally, reference lists of all relevant articles were searched for eligible studies and further random searches on google and google scholar were conducted. Searches were periodically repeated until August 30, 2023 to ensure that any new articles were captured. Abstracts of all the studies generated by the search strategy were independently reviewed by two reviewers for eligibility. Full-text report of those that were eligible for inclusion were retrieved and independently assessed by two reviewers for final inclusion into the study. Where the two reviewers disagreed, the disagreements were resolved through consensus.

### Inclusion and exclusion criteria

Studies included for analysis were original interventional studies that examined the efficacy of DRO for tic suppression. These were single group studies that examined the severity of tics before and after DRO intervention. Studies included were those that reported outcome as tic frequency per minute at baseline and after DRO intervention.

### Data extraction

Data extracted from included studies were: (1) The first author; (2) Year of publication; (3) Country of study; (4) Sample size; (5) Mean age and their standard deviations; (6) Gender; (7) Mean YGTSS scores and their standard deviations; (8) Duration of each experiment session; and (9) Outcome measure. Where the mean outcome data were not available, they were calculated from primary datasets. All data were entered in a standard tabular form and evaluated by two reviewers, to generate consensus on data accuracy and integrity.

### Quality assessments

Literature quality of the included studies were evaluated using the NIH quality assessment tool for before-after (Pre-Post) studies with no control group [30]. The tool assesses studies on twelve criteria that enable reviewers to focus on the key concepts required to evaluate the internal validity of a study. Final quality of a study is then rated as “Good”, “Fair”, or “Poor” based on professional judgement of the reviewers as guided by the tool. Quality assessment was conducted by two independent reviewers and then consensus built.

### Statistical analyses

Statistical analysis was conducted using RevMan 5.3 software. Difference between the means of the number of tics/min at baseline and after DRO intervention were calculated for each study by subtracting baseline mean tics/min from post-DRO mean tics/min, and their pooled SD calculated. Standard errors (SE) were then calculated and mean differences summarized as standard mean difference and its 95% confidence interval. Heterogeneity among studies was assessed using the *I*^2^ method. Based on the study designs, random effect meta-analysis was adopted to combine the effect sizes.

## Results

### Characteristics of included studies

**Fig. 1:**
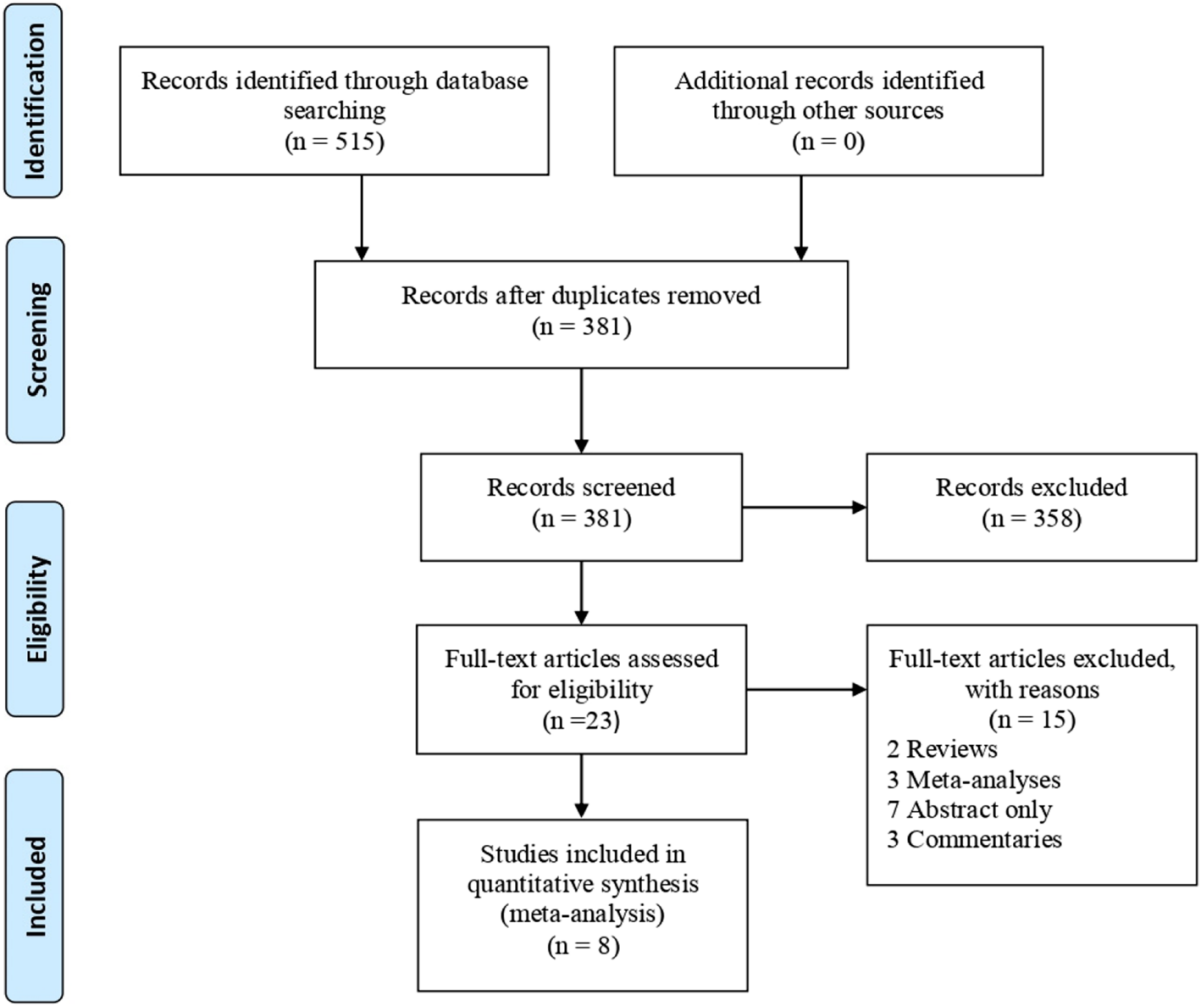
Prisma flow chart for the study selection

A total of five hundred and fifteen studies were identified using the search strategy, of which 8 met the criteria for inclusion into the meta-analysis (Fig. 1). We summarized the characteristics of the included studies in Table 1. The 8 studies (Conelea & Woods, 2008 [15];Greene et al., 2014 [21]; Himle et al., 2007 [31],2008 [28]; Himle & Woods2004 [17]; Specht et al., 2012 [32]; Woods et al., 2007 [33], 2008 [23]) were all Tic intervention studies that employed the DRO technique for tic suppression. They had a combined total of 79 children who met the eligibility criteria.

**Table 1:** Characteristics of included studies

Author	Country	Number of children	Mean Age (SD)	Gender (M/F)	Mean YGTSS (SD)	Duration per session (min)	Mean number of tics/min (SD)	
Conelea & Woods, 2008 [15]	USA	9	11.5 (9–15)	7/2	21.5 (3.6)	6	46.6 (42.8)	5.5 (6.1)
Greene et al. 2014 [21]	USA	21	8.14 (2.79)	14/7	16.6 (6.9)	5	4.4 (3.0)	2.7 (3.1)
Himle et al. 2007 [31]	USA	4	13.5 (2.5)	3/1	25.5 (3.9)	5	16.7 (19.2)	1.8 (1.9)
Himle et al. 2008 [28]	USA	3	9.0 (0.8)	2/1	NA	5	18 (2.8)	4 (2.2)
Himle &Woods, 2004 [17]	USA	7	9.8 (8–12)	6/1	42.3 (11.1)	5	50.0 (20.1)	16.4 (13.7)
Specht et al. 2012 [32]	USA	12	13.8 (2.3)	11/1	27.7 (8.8)	10	13.8 (10.4)	3.2 (2.8)
Woods et al. 2007 [33]	USA	13	10.0 (2.0)	12/1	19.2 (4.1)	5	7.3 (6.3)	3.8 (4.8)
Woods et al. 2008 [23]	USA	10	10.8 (9–15)	10/0	23.0 (6.3)	5	4.29 (3.04)	1.6 (1.7)

*BL* Baseline, *DRO* Differential reinforcement of other behaviors

### Study quality

We used the NIH quality assessment tool for before-after studies with no control group [30]. to assess the quality of the included studies. Supplementary materials 2. Here studies are scored as “Good”, “Fair” or “Poor” based on a list of 12 key criteria that guide the professional judgement of the study assessors. Four of the studies were rated “Good”, while four were rated “Fair”. Key among the 12 criteria were; whether the study participants were representative of those who would be eligible for the intervention, whether all eligible participants were enrolled and whether sample sizes were sufficient enough to give confidence in the findings. Studies that widely advertised in the hospital, social and print media, and were multisite were deemed to have met these criteria. However, sample sizes in all the studies were low. This could be due to the relatively low prevalence of the condition in the study geographical areas.

### Tics severity

**Fig. 2:**
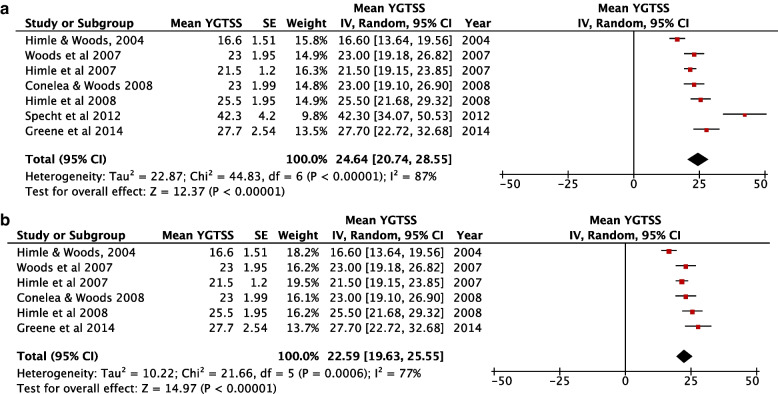
**a** Forest plot for mean YGTSS scores. **b** Forest plot for mean YGTSS scores of only moderate severity tics

Of the 8 studies, one [31] did not report the Yale Global Tic Severity Scale Score (YGTSS) for tic severity among the children. This is a structured clinician-rated scale that scores motor and vocal tics along several dimensions (number, frequency, intensity, complexity, and interference; range, 0 to 5 each). Scores are then summed up as total tic severity score ranging from 0 to 50, where motor and vocal tics are separately scored ranging from 0 to 25 each. The higher the score, the more severe the tic. In this meta-analysis, the combined mean YGTSS score for the remaining 7 studies using the random effect model was 24.64 (95% CI: 21.99 – 30.12, *p* =  < 0.00001, *I*^*2*^ = 87%).), indicating moderate tics among the study participants Fig. 2a. One study assessed children with very high YGTSS scores and could have been the source of the large heterogeneity seen in the meta-analysis. Sub-group analysis excluding that study had a mean YGTSS score of 22.59 (95% CI: 19.63 – 25.55, p =  < 0.00001), and reduced heterogeneity to *I*^*2*^ = 77% Fig. 2b.

### Efficacy of DRO in tic suppression

**Fig. 3:**
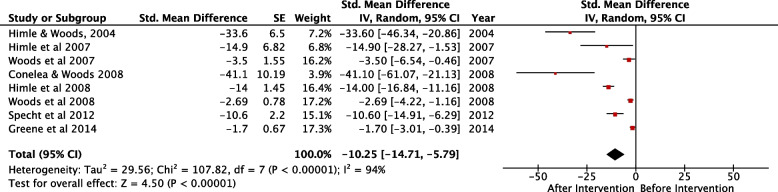
Forest plot for SMD of mean tics/min before and after intervention

All included studies assessed the efficacy of DRO as a technique for tic suppression among children diagnosed with TS/CTD. The combined sample size was 79. Number of tics/min were recorded at baseline (before intervention), and after DRO intervention. This was repeated several times and the mean tics/minute calculated and recorded. Pooled SMD was -10.25 (95% CI: -14.71 – -5.79, *p* =  < 0.00001) with *I*^*2*^ = 94% Fig. 3.

## Discussion

The purpose of the current study was to determine the efficacy of DRO intervention in suppressing tics. All the available studies on DRO intervention in tic disorders involved just a handful of subjects, limiting the statistical power and generalizability of results. We thus combined all these studies in a meta-analysis and generated a combined effect size to enable a more robust effect size determination. Meta-analysis of the number of tics per minute before and after a DRO intervention was conducted from 8 recruited studies. The pooled SMD was -10.25 (95% CI: -14.71 – -5.79) indicating a significant reduction in the number of tics per minute from baseline readings after a DRO intervention. This suggests that DRO is an effective technique for tics suppression. We characterized the strength of the evidence as relatively weak since all the studies were single group studies without controls and none was a randomized controlled trial (RCT). However, the evidence shows that DRO is effective in tic suppression, and sets the basis for future large scale RCTs on this technique in tic management.

In all the 8 studies in this meta-analysis, the DRO interventions involved promising the child a monetary reward for successful tic suppression. To enable replicability in real world settings, it would be ideal to explore the child’s inner fears that exacerbate tic occurrence such as teasing by colleagues, or stares by the public, and use the promise of avoiding such experience as a reward in the experiment [16]. This will ensure a sustainable solution as opposed to monetary rewards. It will also help clarify the fear that DRO could induce satiation as suggested by [34]. For instance, if the child targeted a specific amount of monetary reward and now feels like he/she does not need more reward, they may stop responding to the therapy. Future carefully designed studies could look into this possibility.

Since DRO does not teach the child any alternative behavior to compensate the undesired one, there is a fear that it may reinforce other socially undesirable behaviors [34, 35], for instance, rewarding a child for tic suppression at a time when he/she is spitting or screaming. This area needs further research to develop creative ways of avoiding this problem without reducing the gains made in tic suppression by the child. Furthermore, DRO is a technically demanding process that requires a person to constantly monitor the behavior of the child for specified time intervals, and then dispense reinforcement. This makes treatment for a group of children at the same time very expensive [34]. Going forward, innovative approaches such as wearable technologies can be employed to set reinforcement schedules and track tic frequency, enabling more accurate measurement of tics in response to real-world situations. That would also enable conducting sessions for multiple children by a single psychologist [36].

In terms of tic severity, the pooled YGTSS score was 24.64 (95% CI: 21.99 – 30.12) suggesting that majority of the children had moderate tic disorders. One study [37] evaluated DRO in children with severe tics (mean YGTSS, 42.3), but still the reduction in the frequency of tics after intervention was comparable to the other studies. This suggests that DRO intervention could be effective for both moderate and severe tics. Indeed, DRO intervention has been shown to be effective in treating other severe behavioral disorders such as severe aggression, disruptive behaviors and self-injurious behaviors among others [38–40]. Further studies are needed to clarify the efficacy of DRO by tic severity.

A thorough literature search did not yield any study that specifically compared the efficacy of DRO to other established behavioral Tics suppression strategies such as Habit Reversal Training (HRT), Comprehensive Behavioral Intervention for Tics (CBIT), Exposure with Response Prevention (ERP). However, favorable characteristics of DRO such as ease of application with limited training makes it a technique worth exploring further. The quality of DRO could benefit from new approaches such as machine learning, computational modeling, and imaging techniques that could be used to link behavioral antecedents and consequences to underlying brain mechanisms [36]. This will facilitate deeper understanding of the technique and better design of future experiments. Similarly, in this era of 5G networks and telemedicine, behavioral scientists could consider conducting remote sessions where the child is in a real-world setting like home or school or in the leisure park. Parents or caretakers of the child can facilitate this session. This will significantly enhance sustainability.

Publication bias could not be determined since the studies were few in number. However, heterogeneity among the effect sizes of the various studies was relatively high. Possible explanations for this could be the difference in severity of tics among the children in the different studies, difference in the length of experiment sessions, and other biological differences among the patients.

While interpreting the results of this study, readers need to be aware of the following limitations: 1) Due to the small overall sample size and all the studies being of western origin, the results of this meta-analysis may not be generalizable [41]. 2) Assessment of publication bias was not possible due to the small number of studies analyzed [42]. 3) All the studies in the meta-analysis were single-arm interventional studies that did not have control groups. In this situation, it may be hard to attribute all the success of the Tic suppression among the children to DRO as the authors could not control possible confounding factors [43]. Future studies with proper control groups are needed to clarify our results. 4) Being a scale-based research, evaluator biases inevitably exist that may have affected rating of the children. 5) Only English language studies were enrolled, therefore future studies involving multiple countries that may not use English for publication will provide better outcome.

## Conclusion

In conclusion, this study demonstrated that DRO could be an effective tic suppression technique for temporarily managing tic disorder. It also demonstrated that DRO could be potentially effective for both moderate and severe tic disorders. In one study, children successfully suppressed tics for longer than the commonly used 10 s, suggesting possible efficacy of the technique for longer periods of time. However, the technique bears certain limitations that could impede its implementation outside of experimental settings. Future studies with well-defined control groups, and designed with session intervals longer than 10 s are needed. This will go a long way in ensuring applicability of the intervention in real world settings.

### Supplementary Information

**Additional file 1.** **Supplementary material 1.**

**Additional file 2.** **Supplementary material 2.**

## Data Availability

The data used to support the findings of this study are included within the article.

## References

[1] American Psychiatric Association. Diagnostic and Statistical Manual of Mental Disorders, Fifth Edition. American Psychiatric Association; 2013. 10.1176/appi.books.9780890425596.

[2] Ramkiran S, Heidemeyer L, Gaebler A, Shah NJ, Neuner I (2019). Alterations in basal ganglia-cerebello-thalamo-cortical connectivity and whole brain functional network topology in Tourette’s syndrome. Neuroimage Clin.

[3] Groenewegen HJ, van den Heuvel OA, Cath DC, Voorn P, Veltman DJ (2003). Does an imbalance between the dorsal and ventral striatopallidal systems play a role in Tourette’s syndrome? A neuronal circuit approach. Brain Dev.

[4] Mink JW (2001). Neurobiology of basal ganglia circuits in Tourette syndrome: faulty inhibition of unwanted motor patterns?. Adv Neurol.

[5] Peterson BS (2001). Neuroimaging studies of Tourette syndrome: a decade of progress. Adv Neurol.

[6] Peterson BS (2003). Basal Ganglia volumes in patients with Gilles de la Tourette syndrome. Arch Gen Psychiatry.

[7] Singer HS (2005). Tourette’s syndrome: from behaviour to biology. Lancet Neurol.

[8] Leckman JF (2002). Tourette’s syndrome. Lancet.

[9] Caligiore D, Mannella F, Arbib MA, Baldassarre G (2017). Dysfunctions of the basal ganglia-cerebellar-thalamo-cortical system produce motor tics in Tourette syndrome. PLoS Comput Biol.

[10] Mink JW (2001). Basal ganglia dysfunction in Tourette’s syndrome: a new hypothesis. Pediatr Neurol.

[11] McCairn KW, Iriki A, Isoda M (2013). Global Dysrhythmia of Cerebro-Basal Ganglia-Cerebellar Networks Underlies Motor Tics following Striatal Disinhibition. J Neurosci.

[12] Leckman JF, Bloch MH, King RA, Scahill L (2006). Phenomenology of tics and natural history of tic disorders. Adv Neurol.

[13] Jw M (2003). The Basal Ganglia and involuntary movements: impaired inhibition of competing motor patterns. Arch Neurol..

[14] Wang Z, Maia TV, Marsh R, Colibazzi T, Gerber A, Peterson BS (2011). The Neural Circuits That Generate Tics in Tourette’s Syndrome. AJP.

[15] Conelea CA, Woods DW (2008). Examining the impact of distraction on tic suppression in children and adolescents with Tourette syndrome. Behav Res Ther.

[16] Conelea CA, Woods DW (2008). The influence of contextual factors on tic expression in Tourette’s syndrome: a review. J Psychosom Res.

[17] Himle MB, Woods DW (2005). An experimental evaluation of tic suppression and the tic rebound effect. Behav Res Ther.

[18] Silva RR, Munoz DM, Barickman J, Friedhoff AJ (1995). Environmental factors and related fluctuation of symptoms in children and adolescents with Tourette’s disorder. J Child Psychol Psychiatry.

[19] Cook CR, Blacher J (2007). Evidence-based psychosocial treatments for tic disorders. Clin Psychol Sci Pract.

[20] Piacentini J (2010). Behavior Therapy for Children with Tourette Disorder: A Randomized Controlled Trial. JAMA.

[21] Greene DJ, Koller JM, Robichaux-Viehoever A, Bihun EC, Schlaggar BL, Black KJ (2015). Reward enhances tic suppression in children within months of tic disorder onset. Dev Cogn Neurosci.

[22] Verdellen CWJ, Hoogduin CAL, Keijsers GPJ (2007). Tic suppression in the treatment of Tourette’s syndrome with exposure therapy: the rebound phenomenon reconsidered. Mov Disord.

[23] Woods DW, Walther MR, Bauer CC, Kemp JJ, Conelea CA (2009). The development of stimulus control over tics: a potential explanation for contextually-based variability in the symptoms of Tourette syndrome. Behav Res Ther.

[24] Iannaccone JA, Jessel J (2021). A translational comparison of contingency-based progressive delay procedures and their effects on contextually appropriate behavior. J Appl Behav Anal.

[25] Miltenberger RG, Fuqua RW (1985). A comparison of contingent vs non-contingent competing response practice in the treatment of nervous habits. J Behav Ther Exp Psychiatry.

[26] Miltenberger RG, Fuqua RW, Woods DW (1998). Applying behavior analysis to clinical problems: review and analysis of habit reversal. J Appl Behav Anal.

[27] Woods DW, Murray LK, Fuqua RW, Seif TA, Boyer LJ, Siah A (1999). Comparing the effectiveness of similar and dissimilar competing responses in evaluating the habit reversal treatment for oral-digital habits in children. J Behav Ther Exp Psychiatry.

[28] Himle MB, Woods DW, Bunaciu L (2008). Evaluating The Role of Contingency in Differentially Reinforced Tic Suppression. J Appl Behav Anal.

[29] Page MJ, et al. The PRISMA 2020 statement: an updated guideline for reporting systematic reviews. BMJ. 2021; n71. 10.1136/bmj.n71. 10.1136/bmj.n71 PMC8005924 33782057

[30] “Study Quality Assessment Tools | NHLBI, NIH”. [Online]. Available: https://www.nhlbi.nih.gov/health-topics/study-quality-assessment-tools. Accessed 18 Aug 2023.

[31] Himle MB, Woods DW, Conelea CA, Bauer CC, Rice KA (2007). Investigating the effects of tic suppression on premonitory urge ratings in children and adolescents with Tourette’s syndrome. Behav Res Ther.

[32] Specht MW (2013). Effects of tic suppression: Ability to suppress, rebound, negative reinforcement, and habituation to the premonitory urge. Behav Res Ther.

[33] Woods DW, et al. Durability, Negative Impact, and Neuropsychological Predictors of Tic Suppression in Children with Chronic Tic Disorder. J Abnormal Child Psychol. 2007; 36 (2):237. Accessed 13 Sep 2023. [Online]. Available. https://www.academia.edu/27438074/Durability_Negative_Impact_and_Neuropsychological_Predictors_of_Tic_Suppression_in_Children_with_Chronic_Tic_Disorder. 10.1007/s10802-007-9173-9 17717739

[34] E. G. Carr and A. Others, “Positive Approaches to the Treatment of Severe Behavior Problems in Persons with Developmental Disabilities: A Review and Analysis of Reinforcement and Stimulus-Based Procedures. Monograph No. 4,” Association for Persons with Severe Handicaps, 7010 Roosevelt Way N, 1990. Accessed 10 Nov 2023. [Online]. Available: https://eric.ed.gov/?id=ED330151

[35] ampersandmktg, “DRA vs DRO - How Differential Reinforcement is Used to Help Change Behaviors,” Achieve Beyond. Accessed 29 Aug 2023. [Online]. Available: https://www.achievebeyondusa.com/the-dl-on-dras-and-dros-using-differential-reinforcement-to-help-change-behaviors/

[36] Iverson AM, Black KJ (2022). Why Tic Severity Changes from Then to Now and from Here to There. J Clin Med.

[37] Woods DW, Himle MB (2004). Creating tic suppression: comparing the effects of verbal instruction to differential reinforcement. J Appl Behav Anal.

[38] Jessel J, Ingvarsson ET (2016). Recent advances in applied research on DRO procedures. J Appl Behav Anal.

[39] Matson JL, Shoemaker ME, Sipes M, Horovitz M, Worley JA, Kozlowski AM (2011). Replacement behaviors for identified functions of challenging behaviors. Res Dev Disabil.

[40] Poling A, Ryan C (1982). Differential- Reinforcement-of-Other-Behavior Schedules: Therapeutic Applications. Behav Modif.

[41] Tipton E, Hallberg K, Hedges LV, Chan W (2017). Implications of Small Samples for Generalization: Adjustments and Rules of Thumb. Eval Rev.

[42] Dalton JE, Bolen SD, Mascha EJ (2016). Publication Bias: The Elephant in the Review. Anesth Analg.

[43] Thiese MS (2014). Observational and interventional study design types; an overview. Biochemia Medica.

