# Supplementary material for: Efficacy of differential reinforcement of other behaviors therapy for tic disorder: a meta-analysis
Source: BMC Neurol. 2024 Jan 2;24:3. doi: 10.1186/s12883-023-03501-2 (PMC10759470; doi:10.1186/s12883-023-03501-2)
Supplement: Supplementary file 1 — Additional file 1. Supplementary material 1. [file 12883_2023_3501_MOESM1_ESM.docx]

**Search strategies**

This meta-analysis was based on systematic searches in multiple data sources:

a. Electronic Databases: PubMed/MEDLINE, Embase, PsycINFO, Cochrane Central Register of Controlled Trials (CENTRAL), and other relevant databases.

c. Reference List: Reference lists of included studies and relevant systematic reviews.

Repeat search was conducted at every interval of the study process until the final report was written, to ensure that any new eligible study is immediately recruited.

**URL to search strategy**

PubMed Search Strategy:

((("tic disorders"[MeSH Terms] OR "tic disorders"[Title/Abstract] OR "tourette syndrome"[MeSH Terms] OR "tics"[MeSH Terms]) AND "tic Suppression"[Title/Abstract]) OR "tic Suppressibility"[Title/Abstract] OR "tic control"[Title/Abstract] OR "tic reduc*"[Title/Abstract]) AND "child*"[MeSH Terms]

**Differential Reinforcement of Other Behaviors therapy**

(((("tic disorders"[MeSH Terms] OR "tic disorders"[Title/Abstract] OR "tourette syndrome"[MeSH Terms] OR "tics"[MeSH Terms]) AND "tic Suppression"[Title/Abstract]) OR "tic Suppressibility"[Title/Abstract] OR "tic control"[Title/Abstract] OR "tic reduc*"[Title/Abstract]) OR (Differential Reinforcement of Other Behaviors therapy[MeSH Terms]) AND "child*"[MeSH Terms])

**Detailed Search strategy:**

((("tic disorders"[MeSH Terms] OR "tic disorders"[Title/Abstract] OR "tourette syndrome"[MeSH Terms] OR "tics"[MeSH Terms]) AND "tic Suppression"[Title/Abstract]) OR "tic Suppressibility"[Title/Abstract] OR "tic control"[Title/Abstract] OR "tic reduc*"[Title/Abstract] OR ((("cell differentiation"[MeSH Terms] OR ("cell"[All Fields] AND "differentiation"[All Fields]) OR "cell differentiation"[All Fields] OR "differentiated"[All Fields] OR "differentiation"[All Fields] OR "differential"[All Fields] OR "differentials"[All Fields] OR "differentiate"[All Fields] OR "differentiates"[All Fields] OR "differentiating"[All Fields] OR "differentiational"[All Fields] OR "differentiations"[All Fields] OR "differentiative"[All Fields]) AND ("reinforce"[All Fields] OR "reinforced"[All Fields] OR "reinforcement, psychology"[MeSH Terms] OR ("reinforcement"[All Fields] AND "psychology"[All Fields]) OR "psychology reinforcement"[All Fields] OR "reinforcement"[All Fields] OR "reinforcements"[All Fields] OR "reinforcer"[All Fields] OR "reinforcer s"[All Fields] OR "reinforcers"[All Fields] OR "reinforces"[All Fields] OR "reinforcing"[All Fields]) AND "Other"[All Fields] AND ("behavior"[MeSH Terms] OR "behavior"[All Fields] OR "behavioral"[All Fields] OR "behavioural"[All Fields] OR "behavior s"[All Fields] OR "behaviorally"[All Fields] OR "behaviour"[All Fields] OR "behaviourally"[All Fields] OR "behaviours"[All Fields] OR "behaviors"[All Fields] OR "pattern"[All Fields] OR "pattern s"[All Fields] OR "patternability"[All Fields] OR "patternable"[All Fields] OR "patterned"[All Fields] OR "patterning"[All Fields] OR "patternings"[All Fields] OR "patterns"[All Fields])) AND "therapeutics"[MeSH Terms])) AND "child*"[MeSH Terms].

A total of 85 studies were retrieved from PubMed:

https://pubmed.ncbi.nlm.nih.gov/?term=%28%28%28%28%22tic+disorders%22%5BMeSH+Terms%5D+OR+%22tic+disorders%22%5BTitle%2FAbstract%5D+OR+%22tourette+syndrome%22%5BMeSH+Terms%5D+OR+%22tics%22%5BMeSH+Terms%5D%29+AND+%22tic+Suppression%22%5BTitle%2FAbstract%5D%29+OR+%22tic+Suppressibility%22%5BTitle%2FAbstract%5D+OR+%22tic+control%22%5BTitle%2FAbstract%5D+OR+%22tic+reduc%2A%22%5BTitle%2FAbstract%5D%29+OR++%28Differential+Reinforcement+of+Other+Behaviors+therapy%5BMeSH+Terms%5D%29+AND+%22child%2A%22%5BMeSH+Terms%5D%29&size=200&ac=no&sort=relevance

**Embase Search Strategy:**

(('tic'/exp OR 'tic' OR 'gilles de la tourette syndrome'/exp OR 'gilles de la tourette syndrome' OR 'chronic tic disorder'/exp OR 'chronic tic disorder') AND ('suppression'/exp OR 'suppression') OR 'suppressibility' OR 'tic control') AND ('child'/exp OR 'child')

<https://www.embase.com/#advancedSearch/resultspage/history.1/page.1/200.items/orderby.date/source>.

A total of 197 studies were retrieved from Embase:

**PsycINFO**

Search Alert: "tic disorders in children OR tourette syndrome OR tics AND tic Suppression OR tic Suppressibility OR tic control AND child

<https://web.p.ebscohost.com/ehost/resultsadvanced?vid=23&sid=e06a95d2-91c9-46fd-bbb9-a6176084c6c7%40redis&bquery=tic+disorder+AND+tic+suppression&bdata=JmRiPXBzeWgmZGI9cGRoJmRiPXBiaCZkYj1hOWgmZGI9ZXJpYyZ0eXBlPTEmc2VhcmNoTW9kZT1BbmQmc2l0ZT1laG9zdC1saXZl>

A total of 87 studies were retrieved from Embase:

**Cochrane Central Register of Controlled Trials Search Strategy**

Search Hits

(tic disorders): ti,ab,kw OR (tourette syndrome):ti,ab,kw AND (tic Suppression):ti,ab,kw OR tic Suppressibility OR tic control AND (children):ti,ab,kw

A total of 146 studies were retrieved from Cochrane Central Register of Controlled Trials:
